# Emergence of *Staphylococcus argenteus* in pediatrics: Molecular insights from a hospital in East China

**DOI:** 10.1080/21505594.2024.2396477

**Published:** 2024-08-26

**Authors:** Chao Fang, Zheng Zhou, Jianping Li, Mingming Zhou

**Affiliations:** Department of Clinical Laboratory, Children’s Hospital, Zhejiang University School of Medicine, National Clinical Research Center for Child Health, Hangzhou, Zhejiang, China

**Keywords:** Bloodstream infections, Infant, *Staphylococcus argenteus*, molecular epidemiology

## Abstract

*Staphylococcus argenteus* is a novel species within the *Staphylococcus aureus* complex and can cause serious bloodstream infections (BSIs) in humans, which have been mainly reported in adults, especially the elderly. In this study, we analyzed the molecular characterization of a strain of *S. argenteus* (22WJ8192) isolated from the peripheral vein blood sample of a seven-month-old female infant in Eastern China. The 22WJ8192 belonged to sequence type (ST)2250 and harbored six antibiotic-resistance genes and 53 virulence genes and was resistant to penicillin. Additionally, we conducted a comparative analysis of the molecular characteristics of *S. argenteus* sourced from various origins within the dataset, predominantly from the National Center for Biotechnology Information Collection (NCBI) genome database. Antibiotic-resistance genes *blaR1*, *blaI_of_Z*, *blaZ*, *fosB-Saur*, *tet(L)*, *aph(3”)-IIIa*, *mecA*, and *dfrG* were more prevalent among the strains of human origin. Virulence genes *lukF-PV*, *sak*, *sdrE*, *scn*, *sdrC*, and *sdrD* were more prevalent among strains of human origin. The presence of antibiotic-resistance genes *blaR1*, *blaI_of_Z*, *blaZ*, *fosB-Saur*, and *aph(3”)-IIIa* in strain 22WJ8192 was also more common among strains of human origin in the dataset. Conversely, the antibiotic-resistance genes *tet(L)*, *mecA*, and *dfrG*, typically found in strains of human origin, were not detected in 22WJ8192. Additionally, virulence genes *lukF-PV*, *sak*, *sdrE*, *scn*, *sdrC*, and *sdrD* present in 22WJ8192 exhibited a higher prevalence among strains of human origin in the dataset. In conclusion, this study emphasizes the potential of *S. argenteus* ST2250 to induce severe bloodstream infections in infants, shedding light on the molecular characteristics of this strain.

## Introduction

*Staphylococcus argenteus* is a recently distinguished staphylococcus species and is a genetically differentiated lineage of *Staphylococcus aureus* that received a formal taxonomic classification in 2015 [1]. *S. argenteus* lacks genes that produce the carotenoid botryxanthin, which gives *S. aureus* its characteristic golden color, and *S. argenteus* consequently displays a white colony form on a chocolate AGAR plate [2]. From currently known reports, *S. argenteus* has been shown to cause foodborne illness, infective endocarditis, and skin and soft tissue, bone and joint, and bloodstream infections (BSIs) [3–8]. Most cases of BSIs caused by *S. argenteus* have been reported in adults, especially the elderly [9,10]. In August 2022, a strain of *S. argenteus* (22WJ8192) was isolated from the peripheral vein blood sample of a 7-month-old female infant in Eastern China. This is the first reported case in China and an indication that *S. argenteus* can also cause BSIs in children.

In this study, we analyzed the clinical and molecular characteristics of the 22WJ8192 strain of *S. argenteus*. We collected the genome sequences of *S. argenteus* from the National Center for Biotechnology Information Collection (NCBI) genome database. The collected genome sequences were combined with the sequences of the isolated *S. argenteus* 22WJ8192 strain into a dataset, and the molecular characteristics of *S. argenteus* from different sources were analyzed. Subsequently, a comparison was conducted between the molecular features of 22WJ8192 and the dataset analysis results.

## Materials and methods

### Strains

In August 2022, a strain of *S. argenteus* (22WJ8192) was isolated from a peripheral vein blood sample of a 7-month-old female infant at the Children’s Hospital, Zhejiang University School of Medicine in Hangzhou City. *S. argenteus* was also isolated from a blood sample taken via the peripheral venous catheter (22QT9308) and an ascites sample (22WJ8195) of the female infant. The strains were initially screened by matrix-assisted laser desorption – ionization time-of-flight mass spectrometry (MALDI-TOF MS; Bruker Daltonik, Bremen, Germany; Database: Bruker MBT 9607MSP) and subsequently reconfirmed by whole genome sequencing (WGS) as follows. DNA of the strain was extracted using the QIAGEN-QiaAmp DNA Mini kit (QIAGEN, Hilden, Germany), and then the purity and concentration of extracted DNA were evaluated by BioDrop mLite+ (BioDrop, Cambridge, UK). The Illumina HiSeq X-Ten platform (Illumina, San Diego, USA) was used for WGS. Sequencing reads were trimmed and assembled *de novo* into contigs using the Unicycler pipeline v0.5.0 (https://github.com/rrwick/Unicycler) [11,12].

Antimicrobial susceptibility testing of this strain was performed using Vitek2 Compact (bioMérieux, Marcyl’Étoile, France) and annually published Clinical and Laboratory Standards Institute breakpoints. In addition, relevant information was gathered from medical and laboratory records. Genome sequences of *S. argenteus* from different regions of the globe were downloaded from the NCBI genome database. The genome sequences collected were integrated with those of 22WJ8192 to create a comprehensive dataset for analyzing the molecular attributes of *S. argenteus* from various origins.

### WGS analysis

Gene annotation of all genome sequences was performed using the Prokka pipeline v1.14.6 (https://github.com/tseemann/prokka) [13]. The BLAST Ring Image Generator v0.95 (https://sourceforge.net/projects/brig) [14] was used to generate the circle of genomic comparisons of 22WJ8192, 22QT9308, and 22WJ8195. Multilocus sequence typing (MLST) analysis was performed using the MLST pipeline v2.23.0 (https://github.com/tseemann/mlst) [15]. Antibiotic resistance and virulence genes were detected using ABRicate v1.0.1 (https://github.com/tseemann/abricate) [16]. Core-genome alignment and single-nucleotide polymorphism were analyzed using the Parsnp V1.7.4 (https://harvest.readthedocs.io/en/latest/content/parsnp.html) [17] program included in the Harvest suite, and a phylogenetic tree was constructed. The phylogenetic tree was edited and visualized using iTOL v6 (https://itol.embl.de/) [18].

### Ethical approval

This study was approved by the ethics committee of the Children’s Hospital, Zhejiang University School of Medicine, China (2021-IRB-031).

### Data analysis

IBM SPSS Statistics for Windows version 23.0 (IBM Corp, Armonk, NY, USA) was used for data analysis. Quantitative variables were expressed as the median and interquartile range (25^th^ and 75^th^ percentiles). Differences between categorical variables were analyzed using the chi-square test. Significance was defined as *p* < 0.05, and all tests of significance were two-sided.

## Results

### Clinical and molecular characterization of *S. argenteus*-causing infant BSIs

**Figure 1.**
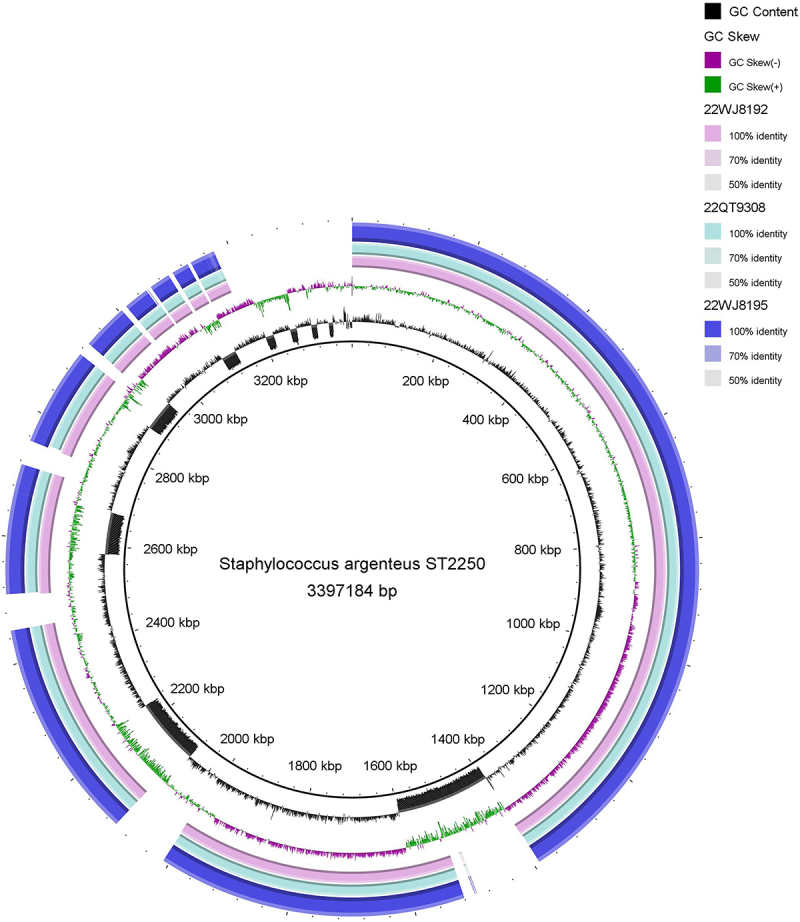
Genomic comparison of 22WJ8192, 22QT9308, and 22WJ8195.

The time to positivity (TTP) of the *S. argenteus* strains 22WJ8192 and 22QT9308 isolated from the infant was 13 h 42 min and 13 h 24 min, respectively; *S. argenteus* was also isolated from the infant’s ascites sample (22WJ8195) 48 h later. By comparing the whole genome sequences of 22WJ8192, 22QT9308, and 22WJ8195, we found that the identity of 22QT9308 and 22WJ8195 was approximately 100% compared with that of 22WJ8192. Therefore, these three strains should have the same origin. The genomic comparison of 22WJ8192, 22QT9308, and 22WJ8195 is shown in Figure 1. We used 22WJ8192 as the representative to analyze the molecular characterization of *S. argenteus*-causing infant BSIs. The *S. argenteus* isolates belonged to ST2250, and the seven housekeeping genes were *arc* (151), *aroE* (325), *glpF* (215), *gmk* (34), *pta* (175), *tpi* (180) and *yqil* (169). This strain harbored 6 antibiotic-resistance genes (no *mecA* gene) and 53 virulence genes and was resistant to penicillin (harbored the *blaZ* gene). The clinical and molecular characterization of *S. argenteus* ST 2250-causing BSIs in infants is shown in Table 1.

**Table 1.** Clinical and molecular characterization of *Staphylococcus argenteus* ST 2250 that caused a bloodstream infection in a child in Eastern China.

Demographic characteristics	
Isolation year	August 2022
Age in months	7
Gender	Female
Underlying diseases	Neuroblastoma
Type of infection	Hospital-acquired
Antimicrobial susceptibility (MIC, μg/mL)	
Penicillin	≥0.5
Oxacillin	0.5
Sulfamethoxazole/trimethoprim	≤0.5/9.5
Clindamycin	0.25
Erythrocin	≤0.25
Ceftaroline	0.25
Rifampicin	≤0.5
Daptomycin	0.25
Linezolid	2
Vancomycin	1
Gentamicin	≤0.5
Moxifloxacin	≤0.25
Levofloxacin	≤0.12
Teicoplanin	4
Resistance gene	
Resistance beta-lactam	*blaZ, blaI_of_Z, blaR1,*
Resistance amikacin	*aph(3’)-IIIa*
Resistance tetracycline	*tet(38)*
Resistance fosfomycin	*fosB-Saur*
Virulence gene	
Ser-Asp rich fibrinogen-binding bone sialoprotein-binding protein	*sdrC, sdrD, sdrE*
Staphostatin B	*sspC*
Staphopain cysteine proteinase SspB	*sspB*
Serine protease	*sspA*
Iron-regulated surface determinant protein	*isdC, isdD, isdE, isdG*
NPQTN specific sortase	*srtB*
Alpha-Hemolysin precursor	*hly/hla*
Panton-Valentine leukocidin chain F precursor	*lukF-PV*
IgG-binding protein SBI	*sbi*
Hemolysin genes	*hlgA, hlgC, hlgB, hlb, hld*
Fibronectin-binding protein	*fnbB, fnbA*
Zinc metalloproteinase aureolysin	*aur*
Related to intercellular adhesion	*icaR, icaA, icaB, icaC, icaD*
Triacylglycerol lipase precursor	*lip*
Adenosine synthase A	*adsA*
Capsular polysaccharide synthesis enzyme	*cap8A, cap8B, cap8C, cap8D, cap8E, cap8F, cap8G, cap8H, cap8I, cap8J, cap8K, cap8L, cap8M, cap8N, cap8O, cap8P*
Type VII secretion system protein	*esxA, esaA, essA, esaB, essB*
Hyaluronate lyase precursor	*hysA*
Complement inhibitor SCIN	*scn*
Staphylokinase precursor	*sak*

### Comparative analysis of the molecular characteristics of S. argenteus from different sources in the dataset

As of 6 July 2023, 217 whole genome sequences of *S. argenteus* that could be distinguished from human and nonhuman sources were retrieved from the NCBI database. Of these, 165 were from humans, and the remaining 52 were from nonhuman sources (mainly from food, animals and the environment). The 22WJ8192 genome sequence was then added to this collection of 217 genome sequences. We subsequently compared and analyzed the differences in the molecular characteristics of *S. argenteus* of human and nonhuman origin. We then traced 166 genome sequences from humans (including 22WJ8192) to 70, where the origin of the specimen could be identified. The 70 genome sequences comprised 10 isolated from blood and 60 isolated from other specimens (mainly from skin, respiratory tract and traumatic wound). We then compared and analyzed the differences in the molecular characteristics of *S. argenteus* isolated from blood and other samples.

#### Human versus nonhuman

**Figure 2.**
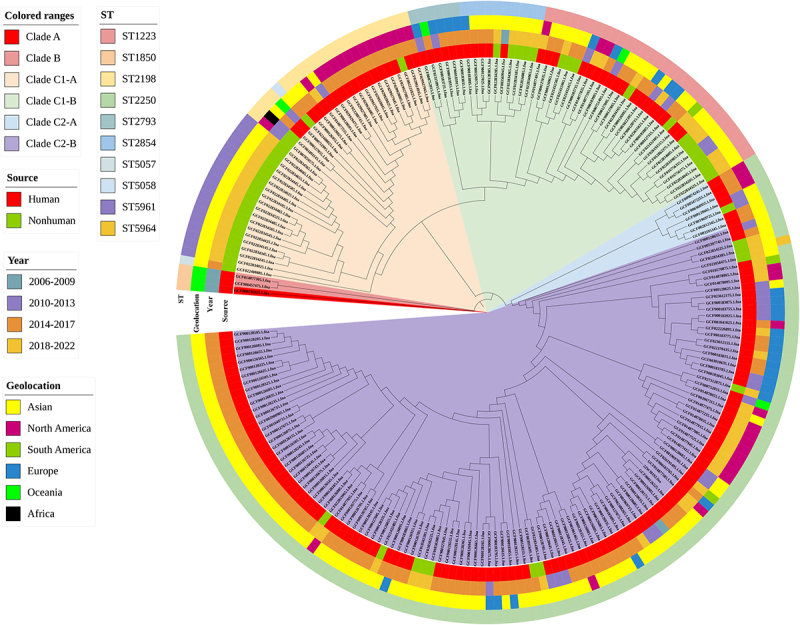
The phylogenetic relationship of 166 genome sequences of *S. argenteus* from humans and 52 genome sequences of *S. argenteus* from nonhuman sources.

A total of 108 (65.1%) of 166 genome sequences from humans were isolated between 2014 and 2017. This was followed by 26 (15.7%) and 27 (16.3%) isolates from 2010–2013 and 2018–2022, respectively. Forty (76.9%) of the 52 genome sequences from nonhuman sources were isolated between 2018 and 2022. In terms of geographical distribution, most strains of human origin were isolated from Asia (93; 56.0%), North America (34; 20.5%), and Europe (29; 17.5%). Most strains of nonhuman origin were also isolated from Asia (48; 92.3%); isolates from other areas accounted for < 10%. MLST analysis showed that 70.0% (116/166) of genome sequences from humans were ST2250, which was significantly higher than 21.0% (11/52) from nonhuman genome sequences (*p* < 10^−3^). The general distribution characteristics of *S. argenteus* strains of human and nonhuman origin are shown in Figure 2.

**Figure 3.**
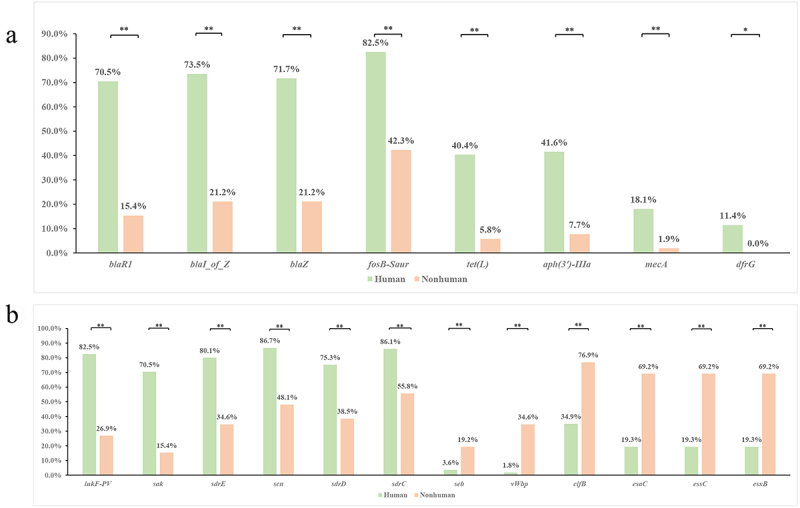
Comparative analysis of antibiotic-resistance and virulence genes of *S. argenteus* isolated from human and nonhuman sources (**: *p* < 0.01; *: *p* < 0.05).

The median antibiotic-resistance gene content of genome sequences from humans was 6.0 (3.0–7.0), which was significantly higher than that of nonhuman genome sequences [1.0 (1.0–2.0), *p* < 10^−3^]. The detection rate of antibiotic-resistance genes *blaR1*, *blaI_of_Z*, and *blaZZ* was > 70% in genome sequences from humans but was < 25% in genome sequences from nonhuman sources (*p* < 10^−3^). The detection rate of the antibiotic-resistance gene *fosB-Saur* reached 82.5% in genome sequences from humans but was only 42.3% (*p* < 10^−3^) in genome sequences from nonhuman sources. The antibiotic-resistance genes *tet(L)*, *aph(3’)-IIIa*, *mecA*, and *dfrG* also were also more prevalent among the strains of human origin. A comparative analysis of the antibiotic-resistance genes of *S. argenteus* isolated from human and nonhuman samples is shown in Figure 3a. The median virulence gene content of genome sequences from humans was 53.0 (52.0–54.0), which was not significantly different from that of nonhuman genome sequences [52.0 (52.0–54.0), *p* = 0.0674]. The detection rate of the virulence genes *lukF-PV*, *sak*, *sdrE*, *scn*, and *sdrD* was > 70% in genome sequences from humans but 50% in genome sequences from nonhuman sources (*p* < 10^−3^). The detection rate of the virulence gene *sdrC* reached 86.1% in genome sequences from humans but was only 55.8% (*p* < 10^−3^) in genome sequences from nonhuman sources. The detection rate of the virulence genes *clfB*, *esaC*, *essC*, and *esxB* was approximately 70% in genome sequences from nonhuman sources but was < 40% in genome sequences from humans (*p* < 10^−3^). The detection rates of virulence genes *seb* and *vWbp* were 19.2% and 34.6%, respectively, in genome sequences from nonhuman sources but were only 3.6% and 1.8% from humans, respectively (*p* < 10^−3^). A comparative analysis of the virulence genes of *S. argenteus* isolated from human and nonhuman sources is shown in Figure 3b.

According to the phylogenetic analysis, the 218 genome sequences of *S. argenteus* could be divided into three clades: A, B, and C. The largest branch is clade C, which includes clades C1 and C2. Clade C1 was divided into clades C1-A and C1-B, and clade C2 was divided into clades C2-A and C2-B. Genome sequences of *S. argenteus* from humans were mainly concentrated in clade C2-B, whereas genome sequences of *S. argenteus* from nonhuman sources were mainly concentrated in clade C1-A. The phylogenetic relationship of 166 genome sequences of *S. argenteus* from humans and 52 genome sequences of *S. argenteus* from nonhuman sources is shown in Figure 2.

#### Blood versus other specimens

**Figure 4.**
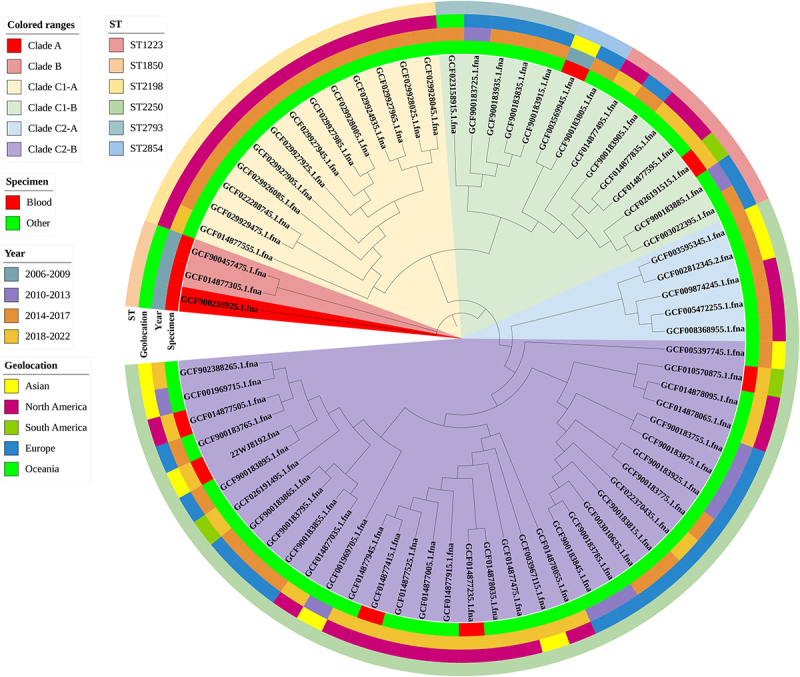
The phylogenetic relationship of 70 isolates that could identify the origin of the specimen of *S. argenteus.*

A total of 6 (60.0%) out of 10 genome sequences from blood were isolated between 2018 and 2022. This was followed by 4 (40.0%) isolates from 2006 to 2009. A further 33 (55.0%) of the 60 genome sequences from other specimens were isolated between 2014 and 2017. This was followed by 18 (30.0%) and 9 (15.0%) isolates from 2018 to 2022 and 2010 to 2013, respectively. The geographic distribution of genome sequences from blood shows a worldwide scattered distribution (Asian: 2, 20.0%; North America: 3, 30.0%; South America: 2, 20.0%; Oceania: 3, 30.0%). Most strains of other specimen origin were isolated from North America (29; 48.3%), followed by Europe (22; 36.7%) and Asia (7; 11.7%). MLST analysis showed ST2250 accounted for 50.0% (5/10) of genome sequences from blood and 58.0% (35/60) of genome sequences from other specimen origins, which was not significantly different (*p* = 0.882). Notably, ST1850 [blood versus other specimens: 30.0.0% (3/10) versus 0.0% (0/60), *p* = 0.002] was more prevalent among the strains of blood origin. The other ST subtypes showed no significant difference between the strains of blood origin and other specimen origin. The general distribution characteristics of *S. argenteus* strains of blood and other specimens of origin are shown in Figure 4.

The median antibiotic-resistance gene content of genome sequences from blood was 3.5 (2.0–6.0), which was not significantly different from that of genome sequences from other specimen origins [5.0 (2.0–6.0), *p* = 0.6199]. No significant difference was present in the detection rate of all the detected antibiotic-resistance genes between the blood and other specimens. The median virulence gene content of genome sequences from blood was 53.0 (52.0–57.0) and was not significantly different from that of other specimen genome sequences [53.0 (51.0–54.0), *p* = 0.1980]. The detection rate of virulence gene *vWbp* was 30.0% in genome sequences from blood, which was significantly higher than that of other specimen genome sequences (0.0%, *p* = 0.002). However, the detection rate of other virulence genes between the blood and other specimens did not significantly differ.

According to phylogenetic analysis, the 70 strains where the origin of the specimen of *S. argenteus* could be identified were divided into three clades: A, B, and C. The largest branch is clade C, which includes clades C1 and C2. Then, clade C1 was divided into clades C1-A and C1-B, and clade C2 was divided into clades C2-A and C2-B. Genome sequences of *S. argenteus* from blood were mainly distributed in clade A, clade B, clade C1-B, and clade C2-B, where clade C2-B was the most distributed (5; 50.0%). However, the genome sequences of *S. argenteus* from blood were not distributed in either clade C1-A or clade C2-A. The phylogenetic relationship of these 70 strains that could identify the origin of *S. argenteus* specimens from different areas is shown in Figure 4.

#### A comparative analysis of the molecular characteristics of *S. argenteus* sourced from various origins within the dataset

The presence of antibiotic-resistance genes *blaR1*, *blaI_of_Z*, *blaZ*, *fosB-Saur*, and *aph(3’)-IIIa* in strain 22WJ8192 exhibited a higher prevalence among strains of human origin within the dataset. Conversely, antibiotic-resistance genes *tet(L)*, *mecA*, and *dfrG*, typically found in strains of human origin, were absent in 22WJ8192. The antibiotic-resistance gene *tet(38)* in 22WJ8192 was universally present across strains of both human and non-human origin in the dataset. Additionally, virulence genes *lukF-PV*, *sak*, *sdrE*, *scn*, *sdrC*, and *sdrD* in 22WJ8192 demonstrated a higher prevalence among strains of human origin in the dataset. The distribution of the remaining 47 virulence genes found in strain 22WJ8192 did not exhibit statistically significant differences between human and non-human strains within the dataset.

## Discussion

*S. argenteus* is a new member of the *S. aureus* complex and has been confirmed by an increasing number of reports as a pathogen of many infectious diseases in humans [19]. BSIs caused by *S. argenteus* have been reported worldwide but are rare and sporadic. Most of the reported BSIs caused by *S. argenteus* cases were among adults, especially the elderly, although *S. argenteus* can also cause infectious diseases in children. For example, a study from Japan reported purulent lymphadenitis caused by *S. argenteus* in a 12-year-old boy [20]. However, BSIs in children caused by *S. argenteus* have not been reported.

In August 2022, a strain of *S. argenteus* was isolated from the peripheral vein blood sample (22WJ8192) of a 7-month-old female infant in our hospital and confirmed by WGS. In addition to the peripheral vein blood sample, *S. argenteus* was also isolated from a blood sample of the patient taken via the peripheral venous catheter (22QT9308) and ascites sample (22WJ8195). 22WJ8192 and 22QT9308 were sampled simultaneously, and the TTP of 22WJ8192 and 22QT9308 were almost the same, with only an 18-min difference. Consequently, the available evidence is inadequate to conclusively classify the infection as catheter-associated. The onset of infection occurred on the 10th day following the child’s admission to the hospital, indicating a hospital-acquired infection with a probable origin of the pathogen within the hospital environment. While the precise mode of transmission remains undetermined, compromised immunity stands out as a significant risk factor for infection in this child (young child and tumor patients). Genome sequence comparison showed that 22WJ8192, 22QT9308, and 22WJ8195 were highly homologous and should be from the same strain. We then analyzed the molecular characteristics of the strain represented by the genome sequence of 22WJ8192. The ST of this strain is ST2250, which is the most common strain of *S. argenteus* [21]. This strain is similar to most *S. argenteus* strains detected worldwide, carries fewer antibiotic-resistance genes, and does not contain the *mecA* gene. This strain contains more of the common virulence genes of *S. argenteus*. Similar to *S. argenteus* isolated from retail foods in China, the biofilm-producing genes *icaA*, *icaC*, *and icaD* and the adhesion genes *fnbB and fnbA* were detected in the genome sequence of the bacterium [22].

We then compared the molecular characteristics of *S. argenteus* sourced from various origins within the dataset. MLST analysis showed that ST2250 was dominant in *S. argenteus* from humans (70.0%) but was less common in *S. argenteus* from nonhuman sources (21%). A study conducted in Japan similarly demonstrated the prevalence of ST2250 among clinical *S. argenteus* [23]. The antibiotic-resistance gene content of *S. argenteus* genome sequences from humans was significantly higher than that of genome sequences from nonhuman sources. This means that *S. argenteus* from humans is more resistant to antibiotics than *S. argenteus* from nonhuman sources. Specifically, antibiotic-resistance genes *blaR1*, *blaI_of_Z*, *blaZ*, *fosB-Saur*, *tet(L)*, *aph(3’)-IIIa*, *mecA*, and *dfrG* were more prevalent among *S. argenteus* from humans. However, no antibiotic-resistance gene has yet been found to be dominant in *S. argenteus* from nonhuman sources compared with *S. argenteus* from humans. Significant differences were also present in the distribution of virulence genes between *S. argenteus* from human and nonhuman sources. The virulence gene *seb* encoding staphylococcal enterotoxin B was more prevalent among *S. argenteus* from nonhuman sources compared with *S. argenteus* from humans. This may be because several of the *S. argenteus* isolates from nonhuman sources were isolated from food specimens, reflecting the potential threat of *S. argenteus* as a foodborne infection. The virulence gene *lukF-PV* encoding leukocidin was more prevalent among *S. argenteus* from humans (82.5%) than from nonhuman sources (26.9%). This may indicate that *lukF-PV* has an important relationship with the pathogenicity of *S. argenteus* in humans. In addition, the virulence genes encoding immune response evasion factors and adhesins were significantly more abundant among *S. argenteus* isolates from humans than from those from nonhuman sources. The virulence genes *sak* and *scn* encoding immune response evasion factors were more common among *S. argenteus* isolates from humans, but the virulence genes *vWbp*, *esaC*, *essC*, and *esxB* encoding immune response evasion factors were more common among *S. argenteus* from nonhuman sources. Analogously, the virulence genes *sdrE*, *sdrC*, and *sdrD* encoding adhesins were more common among *S. argenteus* isolates from humans, whereas the virulence gene *clfB* encoding adhesins was more common among *S. argenteus* from nonhuman sources. Phylogenetic analysis showed that the distribution of *S. argenteus* from humans and *S. argenteus* from nonhuman sources also differed in different evolutionary branches. Most *S. argenteus* isolates from humans belong to Clade C2-B, which is, therefore, closely associated with human infections. Most *S. argenteus* from nonhuman sources belong to Clade C1-A.

Furthermore, an analysis was conducted on the molecular characteristics of human-origin *S. argenteus* obtained from diverse specimens. While MLST analysis showed that ST1850 was dominant in *S. argenteus* isolates from blood, only three strains were present with all coming from the same region at the same time. Therefore, it was not possible to determine whether the ST1850 distribution of *S. argenteus* from blood differed from that of *S. argenteus* from other specimens. Similarly, the virulence gene *vWbp* was also detected in these three strains of *S. argenteus* ST1850. The distribution difference in the virulence gene *vWbp* between *S. argenteus* from blood and *S. argenteus* from other specimens may also be because of geographical factors. Therefore, no meaningful differences in ST or antibiotic-resistance or virulence genes were detected among human-origin *S. argenteus* isolated from different specimens. In addition, the prevalence of ST2250 in *S. argenteus* isolated from blood samples is still dominant, which has also been confirmed in two separate studies conducted in general adult hospitals in China [24,25]. Phylogenetic analysis showed that the distribution of human-origin *S. argenteus* isolated from blood showed a scattered distribution. Although the distribution of *S. argenteus* isolated from blood in branches clade C1-A and clade C2-A was not identified, it is possible that it will appear in the appellate clades as it becomes more widely reported.

Moreover, a comparison was made between the molecular characteristics of 22WJ8192 and the findings of the dataset analysis. The results of the analysis indicated a higher prevalence of antibiotic-resistance genes *blaR1*, *blaI_of_Z*, *blaZ*, *fosB-Saur*, and *aph(3’)-IIIa* in 22WJ8192, which were also commonly found among strains of human origin in the dataset. Consequently, future molecular epidemiological studies focusing on *S. argenteus* isolated from children should prioritize the investigation of these antibiotic-resistance genes. For the same reason, it is imperative that future molecular epidemiological studies of *S. argenteus* isolated from children prioritize the examination of virulence genes *lukF-PV*, *sak*, *sdrE*, *scn*, *sdrC*, and *sdrD*. Given the lack of significant variations in sequence type, antibiotic resistance, and virulence genes among human-derived *S. argenteus* from various specimen sources, the comparison of 22WJ8192 with results from analyses of different specimen sources within the dataset was deemed unnecessary.

It is important to acknowledge a constraint of this study. While the examination of the dataset can elucidate variances in the molecular attributes of diverse origins of S. argenteus to some degree, the compiled genomic information may not entirely capture the genuine epidemic features. The integrity and potential bias of data within the NCBI database are subject to varying degrees of influence due to the diverse origins of data uploads by researchers engaged in various research projects.

The findings of this study underscore the significant threat posed by *S. argenteus* ST2250 in causing severe bloodstream infections in infants, emphasizing the necessity of implementing proactive epidemiological surveillance measures to effectively monitor and mitigate this issue.

## Data Availability

The data that support the findings of this study are openly available in figshare at http://doi.org/10.6084/m9.figshare.25053383.
